# Similarities and Differences in the Acute-Phase Response to SARS-CoV-2 in Rhesus Macaques and African Green Monkeys

**DOI:** 10.3389/fimmu.2021.754642

**Published:** 2021-10-06

**Authors:** Celeste Coleman, Lara A. Doyle-Meyers, Kasi E. Russell-Lodrigue, Nadia Golden, Breanna Threeton, Kejing Song, Genevieve Pierre, Carl Baribault, Rudolf P. Bohm, Nicholas J. Maness, Jay K. Kolls, Jay Rappaport, Joseph C. Mudd

**Affiliations:** ^1^ Department of Immunology and Microbiology, Tulane National Primate Research Center, Covington, LA, United States; ^2^ Department of Microbiology and Immunology, Tulane University School of Medicine, New Orleans, LA, United States; ^3^ Department of Medicine, Tulane University School of Medicine, New Orleans, LA, United States; ^4^ Center for Translational Research in Infection and Inflammation, Department of Pediatrics and Department of Medicine, Tulane University School of Medicine, New Orleans, LA, United States; ^5^ Center for Research & Scientific Computing, Tulane University Information Technology, New Orleans, LA, United States

**Keywords:** COVID-19, systems biology, neutrophil (PMN), interferon, African green monkey

## Abstract

Understanding SARS-CoV-2 immune pathology is critical for the development of effective vaccines and treatments. Here, we employed unbiased serial whole-blood transcriptome profiling by weighted gene network correlation analysis (WGCNA) at pre-specified timepoints of infection to understand SARS-CoV-2-related immune alterations in a cohort of rhesus macaques (RMs) and African green monkeys (AGMs) presenting with varying degrees of pulmonary pathology. We found that the bulk of transcriptional changes occurred at day 3 post-infection and normalized to pre-infection levels by 3 weeks. There was evidence of coordination of transcriptional networks in blood (defined by WGCNA) and the nasopharyngeal SARS-CoV-2 burden as well as the absolute monocyte count. Pathway analysis of gene modules revealed prominent regulation of type I and type II interferon stimulated genes (ISGs) in both RMs and AGMs, with the latter species exhibiting a greater breadth of ISG upregulation. Notably, pathways relating to neutrophil degranulation were enriched in blood of SARS-CoV-2 infected AGMs, but not RMs. Our results elude to hallmark similarities as well as differences in the RM and AGM acute response to SARS-CoV-2 infection, and may help guide the selection of particular NHP species in modeling aspects of COVID-19 disease outcome.

## Introduction

The rapid emergence and dissemination of severe acute respiratory syndrome coronavirus 2 (SARS-CoV-2) and subsequent development of COVD-19 disease has placed an unprecedented burden on the public health system, with greater than 4 million deaths worldwide (https://www.cdc.gov/nchs/nvss/vsrr/covid19/index.htm). The spectrum of SARS-CoV-2 disease severity is heterogenous resulting in asymptomatic to fatal outcomes (1). In subjects with severe COVID-19, disease manifestations include dyspnea, pneumonia, coagulopathy, and lung immune cell infiltration (2, 3). Aged individuals or those with underlying co-morbidities are particularly susceptible to developing severe COVD-19 (4).

Innate immune responses to SARS-CoV-2 play a critical role in COVID-19 disease outcome. Early longitudinal studies have revealed that type I interferon responses are important, with robust yet transient increase of plasma IFNα levels in mild/moderate COVD-19 cases (5, 6), and sustained increases in plasma in IFNα of patients with severe COVID-19 (7). Disease progression is typically characterized by a hallmark cytokine storm, including NF-κB dependent proinflammatory cytokines (IL-1, IL-6, CXCL8, TNF) and chemokines (CCL2, CCL3, CCL4, CXCL10) (7–9). Many of these are likely to influence the trafficking of circulating myeloid cells to sites of SARS-CoV-2 replication, as numerous studies have observed prominent myeloid cell infiltration in the lower airway of subjects with severe COVID-19 (10, 11).

Nonhuman primates (NHP) have been used extensively to model viral pathogenesis and to test therapeutic modalities. Like SARS and MERS (12), an array of NHP species can be infected with SARS-CoV-2 (13–16). The vast majority of experimental infections have resulted in manifestations typical of mild/moderate disease course (13–16), with only rare instances of severe COVID-19 development (17). Nevertheless, it is not completely known whether some NHP species may be better suited than others to model pathogenic features of SARS-CoV-2 infection.

Here, we employed unbiased gene expression profiling by weighted gene network correlation analysis (WGCNA) in blood of rhesus macaques (RMs) and African green monkeys (AGMs) experimentally-infected with SARS-CoV-2. This analysis represents a companion piece to two previously-published studies conducted on the same animals that detail SARS-CoV-2-related immune and histological alterations (17, 18). In brief, SARS-CoV-2 propagated in all animals. Pneumonia of varying grades was observed, concomitant with diffuse alveolar opacities, plasma elevations in a variety of cytokines/chemokines, and migration of myeloid and natural killer (NK) cells into the lung (17, 18). The analysis presented here characterizes dynamics of the acute-phase response in these animals, including defined early timepoints of SARS-CoV-2 infection which have not been as comprehensively investigated in the literature. We show that the bulk of transcriptional alterations in blood of both species occur after 3 days post-infection and normalize to baseline levels by 2 weeks. Gene networks that are coordinated in both species include a generic antiviral response comprised of type I and type II interferons. Pathways associated with neutrophil degranulation were highly enriched in AGMs, yet not observed in RMs. These data elude to hallmark similarities and differences in the acute phase response to SARS-CoV-2 in RMs and AGMs, and may guide the implementation of certain NHP species in modeling particular aspects of COVID-19 disease outcome.

## Results

### WGCNA Reveals Robust Transcriptional Alterations in Blood of SARS-CoV-2 Infected RMs and AGMs

**Figure 1:**
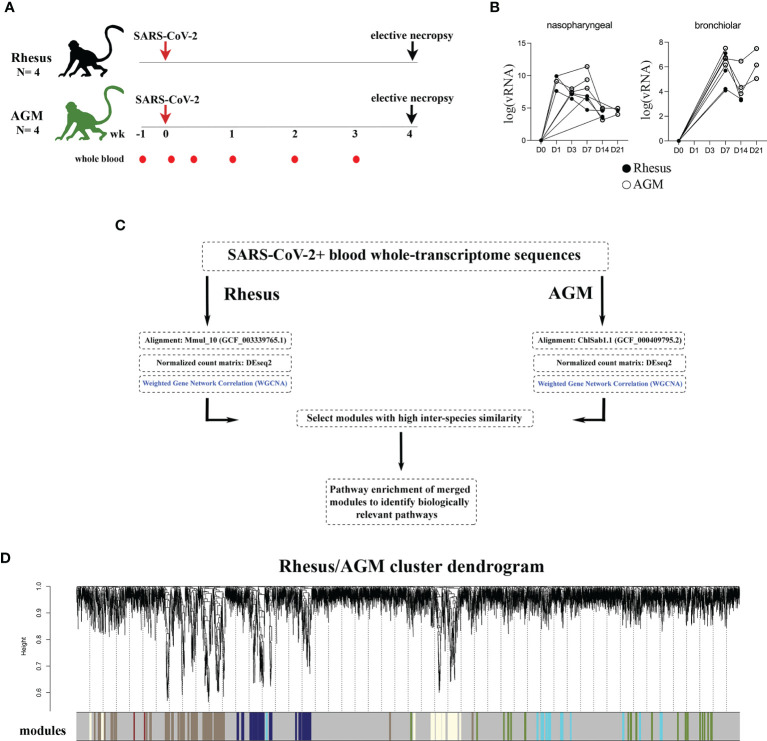
WGCNA reveals robust transcriptional alterations in blood of SARS-CoV-2 infected AGMs and RMs. **(A)** Schematic of study design. Arrows represent SARS-CoV-2 challenge. Red dots represent sampling timepoints of whole-blood RNA sequencing at baseline, 1, 3, 7, 14, and 21 days post-infection. **(B)** SARS-CoV-2 RNA levels in nasopharyngeal and bronchial brushes over time. **(C)** Schematic of computational pipeline employed to uncover groups of genes sharing high inter-species similarity that behaved in a coordinated fashion in response to SARS-CoV-2. **(D)** Clustering dendrogram of genes with dissimilarity based on topological overlap, with gene modules assigned by colors.

Four adult Indian-origin RMs and four adult AGMs were infected with the SARS-CoV-2 isolate USA-WA1/2020 by a multi-route or aerosolized exposure (**Figure 1A** and **Table 1**). To assess host and virological responses throughout infection time course, we sampled blood and the pharyngeal/upper-respiratory tract longitudinally at pre-infection as well as multiple acute and post-acute infection timepoints (**Figure 1A**). At day 28 post-infection the animals underwent elective necropsy. SARS-CoV-2 RNA was detectable in both nasopharyngeal and bronchiolar swabs from all 8 animals in the study. Viral genomic RNA at these sites exhibited similar kinetics to that of SARS-CoV-2+ humans (19, 20), with high levels of viral titers observed in the nasopharyngeal cavity that remained detectable to study endpoint (**Figure 1B**). Although genomic SARS-CoV-2 RNA titers not significantly different between AGMs and RMs or by differing exposure route, animals exhibited a heterogenous response to SARS-CoV-2 infection. Clinical features of these responses are detailed in **Table 1** and illustrated in previous companion studies (17, 18). Notably, 2 AGMs (NC33, NC34) were euthanized prior to study endpoint (day 8 and 22, respectively) due to the development of severe respiratory signs (**Table 1**). Of the remaining 6 that met study endpoint, one AGM (NC38) was clinically asymptomatic, and 4 animals (NC40, GH99, HD09, FR04, HB37) presented with varying grades of interstitial pneumonia upon histopathologic examination. This was severe in GH99, and mild to moderate in NC38, FR04, and HD09 **Table 1**.

**Table 1:** SARS-CoV-2 exposure route, demographic, and clinical outcome of NHPs used in the study.

Animal ID	Exposure (dose)	Route	Age	Sex	Species	Base Weight (kg)	Outcome	Pathological features
GH99	3.61X10^6^ PFU cumulative dose	Multiroute (Oral, intratracheal, intranasal, conjunctival (both eyes	13.9	Male	*Macaca Mulatta*, Indian-orign	15.5	reached study enpoint (27dpi)	severe pnemonia
HD09			12.8	Female	*Macaca Mulatta*, Indian-orign	7.7	reached study enpoint (24dpi)	mild pnemonia
NC40			16 (est)	Male	*Chlorocebus aethiops*	7	reached study enpoint (26dpi)	asymptomatic
NC33			16 (est)	Female	*Chlorocebus aethiops*	3.7	ARDS (22 dpi)	–
HB37	2.0X10^3^ TCID_50_	Aerosol	13	Male	*Macaca Mulatta*, Indian-orign	11.9	reached study enpoint (28dpi)	lymphcytic vasculitis
FR04			15	Male	*Macaca Mulatta*, Indian-orign	12.3	reached study enpoint (28dpi)	minimal pnemonia
NC34			16 (est)	Female	*Chlorocebus aethiops*	4.3	ARDS (8dpi)	–
NC38			16 (est)	Male	*Chlorocebus aethiops*	7.4	reacehd study enpoint (24dpi)	mild/moderate pnemonia

Our overall goal in this study was to unbiasedly uncover gene regulatory networks commonly regulated in SARS-CoV-2 infected AGMs and RMs, particularly at defined acute timepoints where the host response is characterized by only a handful of studies (13–15). Whole blood was subjected to RNA sequencing and resulting reads were aligned to their respective species genomes given that the AGM and RM assemblies and genome annotations are distinct. AGM and RM normalized count matrices were analyzed as separate datasets by Weighted Gene Network Correlation Analysis (WGCNA) to identify gene modules, or groups of genes that behaved in a highly-coordinated fashion in response to SARS-CoV-2 infection (**Figure 1C**). Gene modules were identified by groups of genes that clustered in a hierarchal fashion (**Figure 1D**), comprising 6 modules in the AGM dataset and 8 modules in the RM dataset (**Supplementary Table 1**) between 33 and 1948 genes with a median module size of 575 genes. These data confirm that SARS-CoV-2 specific immune and histological findings observed in previously-reported companion studies of this cohort are also associated with robust transcriptional alterations in blood.

### Particular Modules Exhibit Shared Patterns of Temporal Regulation in SARS-CoV-2 Infected RMs and AGMs

**Figure 2:**
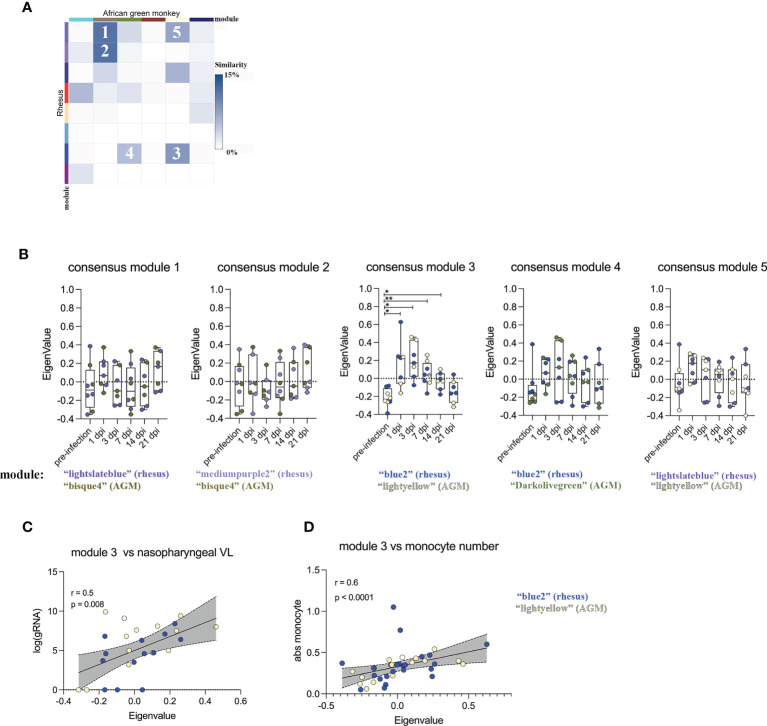
Particular modules exhibit shared patterns of temporal regulation in SARS-CoV-2 infected RMS and AGMs. **(A)** Heatmap representing the percentage of overlap within inter-species pairs of gene modules. Rows and columns represent WGCNA-defined gene modules of RM and AGM, respectively. Coloring sheme represents the degree of inter-species similarity between modules. The 5 inter-species pairs with the highest similarity were ranked 1-5. **(B)** The top 5 module pairs exhibiting high inter-species similarity were merged into "consensus modules" and their module eigenvalue was plotted across the course of SARS-CoV-2 infection. Relationship between the module Eigenvalue of consensus module 3 and **(C)** SARS-CoV-2 gRNA in the nasopharyngeal cavity and **(D)** circulating monocyte number. A matched-pair Wilcoxon rank sum test was used to determine significance in **(B)**. Correlation significance in **(C, D)** were determined by Spearman rank correlation. *p < 0.05, **p < 0.01.

In an effort to identify universal gene expression patterns in the RM and AGM SARS-CoV-2 host response, we ranked modules based on their inter-species similarity by determining the proportion of genes that overlap within each individual species module (**Figure 1C**). Particular inter-species pairs of modules displayed varying degrees of similarity (**Figure 2A**), and we selected 5 of these pairs with the highest proportion of shared genes. These were deemed “consensus modules”, which we hypothesized to contain common genes that are regulated during the generic response to a viral infection (**Figure 2A**). We next determined whether expression levels of genes within each consensus module displayed temporal coordination in response to SARS-CoV-2 by plotting module Eignenvalues across the course of infection. Of the 5 consensus modules, consensus module 3 (comprised of the “blue2 and “light yellow” module of RM and AGM, respectively) behaved in a coordinated fashion in response to SARS-CoV-2, peaking at 3 days post-infection and normalizing to pre-infection levels by day 21 post-infection (**Figure 2B**). Of note, the RM and AGM modules comprising consensus module 3 correlated directly with SARS-CoV-2 genomic RNA in the nasopharyngeal cavity and numbers of monocytes in blood (**Figures 2C, D** **)**, indicating that WGCNA was useful for *a priori* identification of coordinated gene networks that correlated with immune and virological measurements.

### Early Type I Interferon Responses Are a Shared Feature of the SARS-CoV-2 Host Response in RMs and AGMs

**Figure 3:**
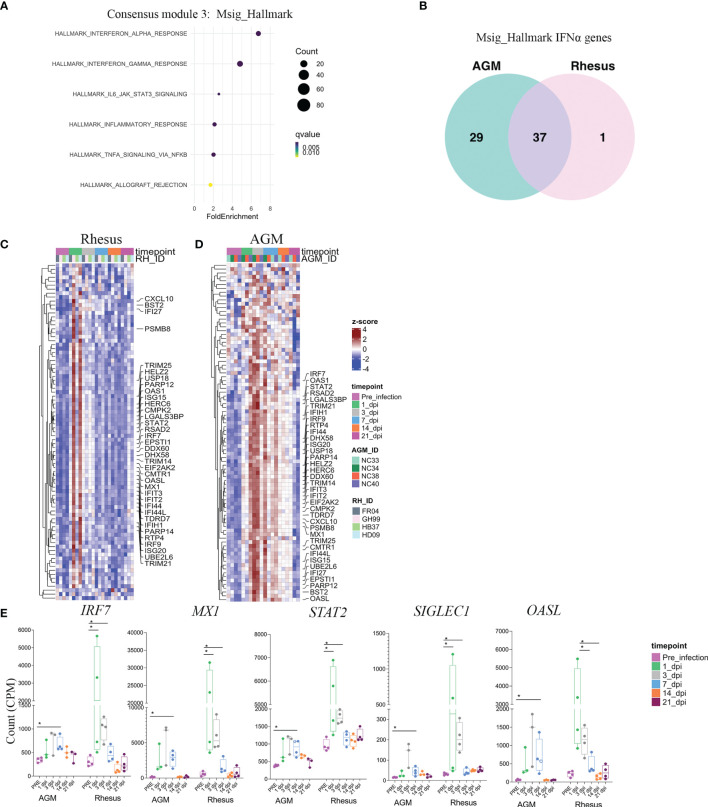
Early type I interferon responses are a shared feature of the SARS-CoV-2 host response in RMS and AGMs. **(A)** Pathway analysis of genes comprised in module 3, according to the Msig_Hallmark pathway database. Fold enrichment represents the ratio of the fraction of consensus module 3 genes Falling with the biological pathway over the fraction of biological pathway genes within the entire gene universe. **(B)** Venn diagram of type I interferon genes comprising consensus module 3 that are shared between species and those that are unique. Heatmaps comprising all genes in the HALLMARK INTERFERON ALPHA pathway are shown in RM **(C)** and AGM **(D)**. Annotated genes represent those within HALLMARK INTERFERON ALPHA present in consensus module 3 that are shared bewteen species. **(E)** Selected genes in the type I IFN response with relavance to SARS-CoV-2 pathogenesis. Significance was assessed by a multiple comparison corrected Fisher’s Exact test in **(A)**, and a non-parametric Mann-Whitney U test in **(E)**. *p < 0.05.

Given our identification of a commonly regulated gene network among SARS-CoV-2 infected animals, we next performed pathway analysis on these genes to determine their biological significance. Enrichment analysis revealed robust representation of a type I interferon gene signature in module 3 (**Figure 3A**). 37 of these interferon-stimulated genes (ISGs) were common to both SARS-CoV-2 infected AGMs and RMs (**Figure 3B** and **Supplementary Table 2**). AGMs displayed an additional 29 genes that were uniquely up-regulated in response to SARS-CoV-2 (**Figure 3B** and **Supplementary Table 2**). In 2 RMs (GH99 and HD09), ISG expression was robustly up-regulated following 1 day post-infection, whereas ISG signatures in blood of AGMs was apparent by day 3 post-infection of all animals (**Figure 3C**). This signature contained a number of genes that are central regulators of the type I interferon response. For example, the gene *IRF7*, a master regulator of the type I IFN response whose loss-of-function associates with life-threatening COVID-19 in human subjects (21, 22), exhibited increased expression in blood as early as day 1 post-infection and normalized by 14 days post-infection (**Figure 3D**). A number of other SARS-CoV-2-induced antiviral genes followed a similar temporal pattern of expression, including known hallmarks of the type I IFN response such as STAT2 and OASL (**Figure 3E**). Notably, expression of the gene encoding SIGLEC1, an interferon inducible cell surface molecule expressed exclusively on myeloid cells (23, 24), was robustly upregulated at 3 days postinfection (**Figure 3E**), suggesting a potentially useful surrogate of the SARS-CoV-2 induced type-I IFN response that can be assessed by routine flow cytometric staining.

### The Acute SARS-CoV-2 Response in Blood Extends to Genes Associated With Type II Interferon Signaling

We further probed the biological pathways enriched in consensus module 3 to determine potential exposure of cytokine networks beyond type I interferons that are indicative of the cytokine storm induced by severe SARS-CoV-2 infection in humans (6). Blood transcriptomes of SARS-CoV-2 infected RMs and AGMs were additionally enriched for an IFNγ gene signature (**Figure 3A**). Many of these genes were found to be overlapping to those present in the type I interferon pathway database. Comparison of the IFNγ gene signatures between species revealed both commonalities and differences in the breadth of the IFNγ-induced genes that were not unlike species-specific comparisons of the type I IFN gene signature. For example, 49 IFNγ-related genes upregulated in consensus module 3 were common to both the RM and AGM SARS-CoV-2 response (**Supplementary Figures 1A–C**). However, AGMs exhibited upregulation of an additional 44 IFNγ-related genes in response to SARS-CoV-2 that were not over-expressed in SARS-CoV-2 infected RMs (**Supplementary Figure 1A**). Notably, one of the genes upregulated in AGMs and RMs that was unique to the IFNγ pathway was Programmed death-ligand 1 (PD-L1) (**Supplementary Figure 1D**), an adaptive immune suppressor induced highly on the surface of myeloid cells and readily detectable by flow cytometric staining. Because type I and type II interferon pathways can overlap significantly, we next determined the proportion of genes that were redundant versus those that were exclusive to type I and type II interferon signaling within RMs or AGMs. Comparison between the two species revealed that most IFN-related genes upregulated by SARS-CoV-2 infection in RM were redundant to both type I and type II interferon signaling (**Supplementary Figure 1E**). In contract, AGMs exhibited greater numbers of genes that were exclusive to the type I or type II interferon pathways (**Supplementary Figure 1E**), highlighting a more defined interferon response in AGMs. In sum, this analysis revealed a transcriptomic response in blood of SARS-CoV-2 infected animals that was dominated by type I and type II interferon-responsive genes, with a greater breadth of these responses exhibited in the AGM.

### Biological Pathways Related to Neutrophil Degranulation Are Unique to the AGM SARS-CoV-2 Response

**Figure 4:**
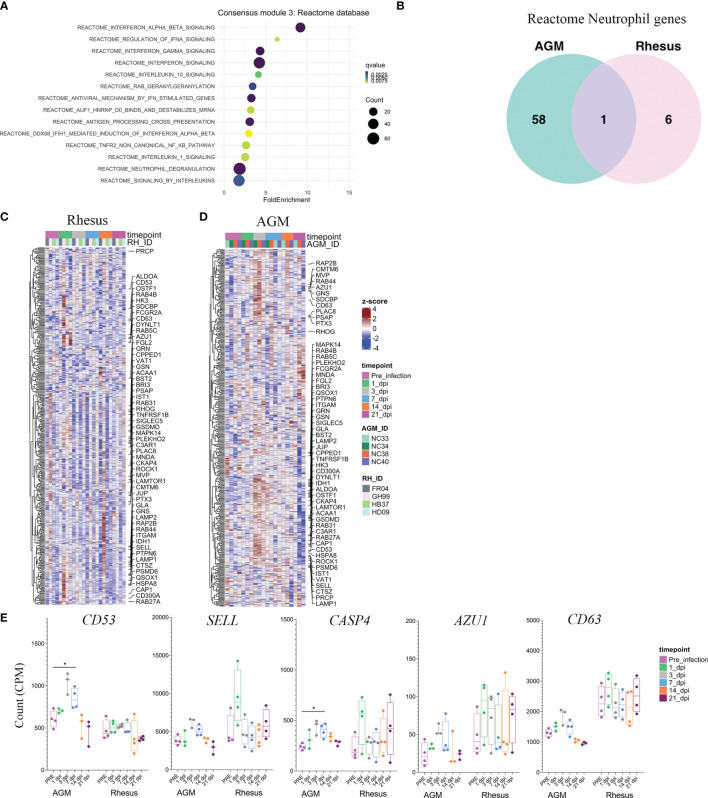
Biological pathways related to neutrophil degranulation are unique to the AGM SARS-CoV-2 response. **(A)** Pathway analysis of genes comprised in consensus module 3 according to the Reactome pathway database. **(B)** Venn diagram of neutrophil degranulation genes comprising consensus module 3 that are shared between species and those that are unique between RM and AGM. Heatmaps comprising all genes in the neutrophil degranulation pathway in RM **(C)** and AGM **(D)**. Annotated genes represent those within the neutrophil degranulation pathway present in consensus module 3 shared bewteen species. **(E)** Selected neutrophil degranulation genes with relavance to SARS-CoV-2 pathogenesis. Significance by a Fisher’s Exact test in **(A)** and a non-parametric Mann-Whitney U test in **(E)**. *p < 0.05.

In addition to the Molecular Signatures (Msig) Hallmark pathway database, we performed enrichment analysis of genes in consensus module 3 against other pathway databases. Enrichment of these genes according to Reactome-curated gene sets yielded similar biological pathways to that of the Msig Hallmark analysis. The most over-represented pathways were those related to type I and type II interferon signaling (**Figure 4A**). Tumor Necrosis Factor (TNF) and IL-1-related signaling pathways were enriched, albeit to a lesser extent (**Figure 4A**). A pathway not included in the Msig Hallmark analysis, but highly significant to the Reactome database was that of neutrophil degranulation (**Figure 4A**). Interestingly, the neutrophil degranulation signature was almost entirely driven by genes in consensus module 3 that were uniquely upregulated in SARS-CoV-2 infected AGMs (**Figure 4B** and **Supplementary Table 2**). Of the 65 neutrophil-related genes in consensus module 3, 58 were unique to AGMs, whereas only 1 gene in the neutrophil pathway was shared between species, and 6 were unique to RMs (**Figure 4B** and **Supplementary Table 2**). Genes related to neutrophil degranulation in blood of AGMs followed a similar temporal pattern to that of the type I/II interferon response, peaking at day 3 post-infection, persisting to day 7, and normalizing to baseline levels by day 14 post-infection (**Figures 4C, D**). This temporal coordination was notably absent in SARS-CoV-2 infected RMs (**Figures 4C, D**). Particular neutrophil-related genes that comprised the AGM SARS-CoV-2 response included *CD63*, a membrane-bound protein strongly expressed on the surface after neutrophil activation and a marker for neutrophil granule release (**Figure 4E**) **(** 25). The gene encoding L-selectin (*SELL*), a prominent neutrophil activation marker important for neutrophil trafficking to inflamed tissues (26) (**Figure 4E**). Transcripts encoding for Azurocidin 1 (*AZU1*) were also upregulated in blood of SARS-CoV-2 infected AGMs, a cationic protein that is localized to neutrophil granules and an important inflammatory mediator with monocyte chemotactic and antimicrobial activity (**Figure 4E**) (27). These results suggest that in this cohort of animals, AGMs exhibited a broader response to SARS-CoV-2 that included additional components of innate immunity such as of neutrophil activation.

## Discussion

Nonhuman primates have long represented an important animal model of viral infections due to their close physiological similarity to humans and the ability to interrogate the host response in greater temporal and anatomical detail. Like other coronaviruses, several studies have shown that a wide range of nonhuman primate species are permissive to SARS-CoV-2 infection (12–18). While the majority of these were conducted on a relatively small number of animals, they have uncovered common characteristics in the host response that supersede species-level differences. These include an early myeloid cell-driven innate immune response accompanied by mild to moderate pneumonia. The vast majority of nonhuman primates experimentally infected with SARS-CoV-2 resolved pulmonary viremia and lung pathology by 2 weeks post infection (12–18). Importantly, these features appear to parallel in many respects mild to moderate disease course in SARS-CoV-2 infected humans (28).

Here, we employed an unbiased transcriptional profiling method in WGCNA to probe early timepoints of the acute response to SARS-CoV-2 in RMs and AGMs. We find that both RMs and AGMs exhibited a systemic type I and type II interferon gene signature that correlated directly with SARS-CoV-2 genomic RNA in the nasopharyngeal cavity and also with numbers of circulating monocytes. In the majority of these animals, expression of interferon-stimulated genes peaked 3 days following SARS-CoV-2 infection and normalized to pre-infection levels by day 14 post-infection. A companion study revealed dramatic pulmonary infiltration of CD16-expressing monocytes that was apparent in these animals 7 days post-SARS-CoV-2 exposure (18). Thus, it is likely that post-peak decay of interferon gene signatures observed in blood of these animals is not entirely reflective of resolution per se, but trafficking of activated myeloid cells to local sites of SARS-CoV-2 replication. The findings of this study taken into context of later, tissue-specific, timepoints of companion pieces illustrate a model whereby local SARS-CoV-2 replication in the upper airway induces circulating monocyte activation, a fraction of which may traffic to the SARS-CoV-2 infected lung and adopt a phenotype in line with more mature, tissue resident macrophages. Several of these animals continued to exhibit immune abnormalities in the lung at day 21 post-infection (18), a time at which blood innate immune signatures resolved. Thus, immune profiles in blood at later timepoints may not entirely reflect the immune dynamic in tissues at later timepoints of infection.

Common to each species was a robust interferon gene signature, with AGMs exhibiting a greater number of ISGs to be upregulated than that of SARS-CoV-2 infected RMs. The interferon gene signature we observe here overlaps significantly with antiviral responses reported for other human respiratory viral infections. Subjects infected with Influenza A virus, Respiratory Syncytial Virus (RSV), human Metapneumovirus (hMPV), various flaviruses and coronaviruses all exhibit interferon gene signatures (29–34). Upstream regulators, such as *IRF3*, *IRF7*, and *STAT1* that we observe to be upregulated in SARS-CoV-2 infected NHPs are also activated by other respiratory viruses (35–37), and the interferon gene signature observed here is likely representative of the generic acute phase response to a respiratory viral infection. The importance of interferons in viral respiratory infections are highlighted by (1) conserved interferon evasion mechanisms of respiratory viruses and (2) the fact that exogenous interferon treatment profoundly affects disease course (38–40). For instance, pegylated IFNα is protective to airway epithelial cells in SARS coronavirus infection in macaques (41). In regard to SARS-CoV-2, the timing and duration of the interferon response is likely a critical factor in disease outcome. We show here that the interferon gene signature in both RMs and AGMs is transient. A short-lived, more coordinated induction of ISGs observed in our study and others may underlie the lack of severe COVID-19 development in SARS-CoV-2 infected NHPs. These are in contrast to some reports of severe COVID-19 in humans in which type I and type II interferon responses are more sustained (5, 7).

We show here that pathways relating to neutrophil degranulation are enriched in the blood of SARS-CoV-2 infected animals. Unlike interferon responses, this signature was driven almost entirely from SARS-CoV-2 infected AGMs. Interestingly, the absolute neutrophil counts in blood did not differ longitudinally in all animals or when animals were stratified by species, although the companion piece to this study revealed marked pulmonary infiltration of CD11b+ neutrophils at later timepoints of SARS-CoV-2 infection, particularly in the AGMs (18). These data suggest pronounced neutrophil activation may be a defining contrast between SARS-CoV-2 infected AGMs and RMs, and that neutrophil responses may identify particular NHP species that respond more pathogenically to SARS-CoV-2. A caveat to this study is that two SARS-CoV-2 infected AGMs developed ARDS and had to be euthanized prior to endpoint. To date, no other studies employing AGMs have reported fatal COVID-19 disease outcomes, and it is possible that other factors such as the frequent anesthesia and sampling regimens employed here could have contributed to ARDS development in these particular instances. Nevertheless, pulmonary neutrophil infiltration has been observed in other instances of AGM SARS-CoV-2 infection (13, 14, 18), and there is ample evidence to suggest neutrophil activation may be an important predictor of COVID-19 disease severity in the clinic. Neutrophilia has been described as an indicator of severe respiratory symptoms in patients hospitalized with COVID-19 (42–44), and neutrophil infiltration in the lower respiratory tract has been observed at autopsies in fatal COVID-19 outcomes (45). The evidence taken together may implicate neutrophils as potential targets for treatment of severe COVID-19. Indeed, SARS-CoV-2 infected RMs treated with the JAK inhibitor Baricitinib exhibited reduced neutrophil infiltration into the lungs and reduced neutrophil extracellular trap (NET) activity (14). Baricitinib is currently being investigated in COVID-19 clinical trials (ACTT-2; NCT04401579) (NCT04421027).

These data highlight critical findings suggested by other studies that NHP species respond differentially to SARS-CoV-2, and that particular NHP species may be better suited to model SARS-CoV-2 pathogenicity than others. While accumulating evidence suggests AGMs may mount a more inflammatory response, the field currently lacks an NHP model of severe COVID-19 disease. The use of aged animals, those with high BMI indexes, or employing particular treatments such as high-fat diets in SARS-CoV-2 infected animals may enhance pathogenicity and prove useful in evaluating therapies aimed at resolving severe incidences of COVID-19 in patients (46). Notably, humoral and cellular adaptive immune responses to SARS-CoV-2 are unambiguously apparent in NHPs, regardless of species (13, 47, 48). Thus, NHPs will continue to prove invaluable in the evaluation of current and future SARS-CoV-2 vaccine candidates.

## Materials and Methods

### Ethical Statement on Animal Use and SARS-CoV-2 Handling

The Institutional Animal Care and Use Committee of Tulane University reviewed and approved all the procedures for this study. The Tulane National Primate Research Center is fully accredited by the AAALAC. All animals were cared for in accordance with the ILAR Guide for the Care and Use of Laboratory Animals 8th Edition. Procedures for sample handling, inactivation, and transfer from BSL3 containment were approved by the Tulane University Biosafety Committee.

### Animals and Infection

Four adult-aged African green monkeys (Caribbean origin) and four rhesus macaques (Macaca mulatta, Indian ancestry) were exposed to SARS-CoV-2; 2019-nCoV/USA-WA1/2020409 (MN985325.1). The virus stock was prepared in Vero E6 cells and the sequence confirmed by PCR and/or Sanger sequencing. Plaque assays were performed in Vero E6 cells. The rhesus macaques were from the Tulane National Primate Research Center breeding colony pathogen-free for simian type D retrovirus (SRV), macacine herpesvirus 1 (B virus), simian immunodeficiency virus (SIV), simian T cell lymphotropic/leukemia virus (STLV), measles virus (MV) and Mycobacterium tuberculosis (TB). The African green monkeys were wild-caught and were kept at the TNPRC for over a year before being assigned to this study. To mimic different possible routes of infection in humans, animals were exposed to the virus either by aerosol (inhaled dose of 2.0 × 10^3^ and 2.5 × 10^3^ TCID_50_) or by inoculating a cumulative dose of 3.61 × 10^6^ PFU through multiple routes (oral, nasal, intratracheal, conjunctival) (**Table 1**).

The multi-route exposure was given to four animals, one adult RM male (GH99, 14 years old), one adult RM female (HD09, 13 years old) and two AGM, one aged male (NC40, 16 years old, approximately) and one aged female (NC33, 16 years old, approximately). An additional two adult male RM (FR04 and HB37, 15 and 13 years old, respectively) and one aged male and female AGM: NC34 and NC38 (16 years old, approximately) were exposed by aerosol) (**Table 1**).

### Blood Collection and RNA Processing

Whole venous blood was collected from nonhuman primates using a BD Vacutainer Safety-Lok Blood Collection Set (BD catg#367281) and BD Vacutainer one use needle holder (BD catg# 364815). Blood was added to the PAXgene Blood RNA reagent tube (PreAnalytiX catg#762165) in the same ratio as the manufacturer’s guidelines. Post collection, PAXgene blood tubes were stored upright for a minimum of two hours and then placed into the -20C freezer until further processing. Samples were processed in accordance with the PreAnalytiX PAXgene Blood RNA Kit (PreAnalytiX catg#762165). The RNA Clean & Concentrator-5 kit (Zymo, Catg#R1015) was used to purify and increase RNA samples with low yields. Procedures were followed per manufacturer’s guidelines. For AGM NC33, Paxgene tubes were not collected at 1 and 3 dpi due to blood being prioritized for other assays.

### Viral RNA Detection

RT-qPCR reaction TaqPath1-Step MultiplexMaster Mix was used (Cat.# A28527, ThermoFisher) along with the 2019-nCoV RUO Kit (Cat.# 10006713, IDTDNA, Coralville, IA) targeting the N1 amplicon of N gene of SARS2-nCoV19 (accession MN908947). The master mix was added to the microtiter plates covered with optical film (Cat.# 4311971; ThermoFisher) and then was vortexed and pulse centrifuged. The RT-qPCR program consisted of incubation at 25°C for 2 min, RT incubation at 50°C for 15 min, and an enzyme activation at 95°C for 2 min followed by 40 cycles of denaturing step at 95°C for 3 s and annealing at 60°C for 30 s. Fluorescence signals were detected with an Applied Biosystems QuantStudio6 Sequence Detector. Data were captured and analyzed with Sequence Detector Software v1.3 (Applied Biosystems, Foster City, CA). Viral copy numbers were calculated by plotting Cq Values obtained from unknown (i.e. test) samples against a standard curve representing known viral copy numbers. The limit of detection of the assay was 10 copies per reaction volume. A 2019-nCoVpositive control (Cat.# 10006625, IDTDNA) were analyzed in parallel with every set of test samples along with a non-template control.

### Bulk RNA Sequencing

Total RNA samples were quantitated using Qubit RNA HS Assay kit (Invitrogen Q32855). A RIN for each sample was determined by running 1ul on an Agilent 4150 TapeStation using RNA Screen Tapes (Agilent 5067-5576). 1-2 ug of each sample were treated with Baseline Zero DNase (Epicentre, DB0711K) for 30 minutes at 37°C. DNase was inactivated by adding 2ul 10X Stop solution and following a 10 minute incubation at 65°C. The Total RNAs were then purified using Agencourt RNAClean XP magnetic beads by following the manufactory recommendation (Beckman Coulter Life Sciences). 10 ng of each sample were used to make SMART-Seq libraries following the SMART-Seq Stranded Kit User Manual (Takara Bio). Briefly, cDNAs were first generated from all total RNA fragments after RNA fragmentation. Addition of Illumina adapters and indexes and then library purification were followed. Final library amplification and purification were performed after enzymatically removing ribosomal fragments originating from rRNA molecules by using probes specific to mammalian rRNA.

Libraries were quantitated using Qubit DNA HS Assay kit (Molecular Probes Life Technologies, Q32854). Average size of each library was determined by running each sample on Agilent 4150 TapeStation using D1000 Screen Tapes (Agilent 5067-5582). Each library was diluted to 4nM in DNase RNase free ultrapure water (Invitrogen 10977-015). All libraries were pooled at a concentration of 4nM before denaturing with 0.2 N NaOH for 5 min at RT. The libraries were neutralized using 0.2 N Tris-HCl, pH 7 before being diluted to 20pM in HT1 buffer (Illumina).

Finally, denatured libraries were loaded into an Illumina NextSeq 550 v2 High-Output reagent cartridge at a final concentration of 1.2pM in HT1 buffer. Denatured PhiX control library was also included at 1% concentration. Dual indexes and paired end 75bp sequencing was performed on a High-Output flow cell yielding about 18M read pairs per sample.

### Bioinformatic Pipeline

Sequencing reads were demultiplexed and adapter-trimmed with Trim Galore (https://www.bioinformatics.babraham.ac.uk/projects/trim_galore/). The –hardtrim option was used to clip the first 6 basepairs of mate R2 that exhibited low quality scores. Because reads were derived from 2 separate species with different genomes and different genome annotations, these were treated as different datasets from alignment to gene correlation steps. In brief, lanes were concatenated and mapped with STAR aligner to their respective species genomes, Mmul_10 (GCF_003339764.) for RM and ChlSabl.1 (GCF_000409795.2) for AGM (49). Parameters were modified from the default run option that gave the highest percentage of uniquely mapped reads: –outFilterScoreMinOverLread 0 –outFilterMatchNmin 0 –outFilterMatchNminOverLread 0 –outFilterMismatchNmax 2. Counts were generated by the STAR option –quantMode geneCounts and an *n* × *n* matrix was prepared as input for the R package *DESeq2* (50). Data was fitted to a generalized linear model in accordance with the DEseqdataset function. Samples that received an RNA concentration step during the isolation process were found to strongly influence fitted values of the model. Thus a revised analysis was performed incorporating the effect of RNA clean-up into the generalized linear model. Hidden variables were further incorporated with the R package *SVA* and the object was post-filtered to remove genes with at least 4 samples having normalized counts < 10. For module analysis, an appropriate soft power threshold was determined to calculate the adjacency matrix. According to the WGCNA tutorial (51), reducing the soft power threshold leads to smaller numbers of modules that are larger in size, whereas increasing the soft power generates a larger number of small-sized modules. To determine the optimal threshold, we ran WGCNA against several soft powers and assessed biological relevance of the generated modules by pathway analysis. We chose the soft power threshold that yielded the greatest number of pathways relevant to a generalized response to viral infection, which was found to be 12. A topology overlap matrix (TOM) was generated at the appropriate soft power and similar modules were merged using a disimillarity threshold of 0.5. The module Eigenvalues, the corresponding genes in each module and how they behaved across time were then used to interpret the SARS-CoV-2 response in AGM and RM. Data pertaining to these were visualized in R with various R packages.

### Statistical Analysis

Significant temporal variation of modules across time was tested using the pair-wise Wilcoxon rank-sum test, followed by *post-hoc* correcting with Dunn’s multiple comparison test. Fold enrichment of biological pathways was determined using the R package ‘ClusterProfiler’ which employs a Fisher’s exact to determine pathway significance, which was manually set against 1000 random permutations. Correlation analysis was performed using the non-parametric Spearman rank correlation method (two-tailed, 95% confidence) using exact permutation P-values when ranks were used. Significance of individual gene expression changes across time were determined using the non-parametric Mann-Whitney U test. All statistical analyses were performed using GraphPad Prism (version 8.4.2 GraphPad Software, La Jolla California USA) and R software (URL: http://www.R-project.org/).

## Data Availability Statement

The data presented in the study are deposited in the NCBI Gene Expression Omnibus (GEO) repository, accession number GSE184949.

## Ethics Statement

The animal study was reviewed and approved by The Institutional Animal Care and Use Committee of Tulane University reviewed and approved all the procedures for this study. The Tulane National Primate Research Center is fully accredited by the AAALAC. All animals were cared for in accordance with the ILAR Guide for the Care and Use of Laboratory Animals 8th Edition. Procedures for sample handling, inactivation, and transfer from BSL3 containment were approved by the Tulane University Biosafety Committee.

## Author Contributions

CC analyzed data and assisted in writing the manuscript. LD-M was the project veterinarian, contributed to study design, and writing of the I.A.C.U.C. KR-L was a project veterinarian, made clinical assessments and collected samples. NG and BT contributed to study design, study coordination, sample processing, and S.O.P. development. CB provided command-line and high performance cluster assistance. JK, KS, and GP oversaw RNA sequencing and library preparation. RB contributed to study design. JR designed and supported the animal study and helped with the writing of the manuscript. JM conceived and supported the study, analyzed data, and wrote the manuscript. All authors contributed to manuscript review. All authors contributed to the article and approved the submitted version.

## Funding

We would like to thank the NIH for supporting this work through the TNPRC base grant (P51 OD011104 59).

## Conflict of Interest

The authors declare that the research was conducted in the absence of any commercial or financial relationships that could be construed as a potential conflict of interest.

## Publisher’s Note

All claims expressed in this article are solely those of the authors and do not necessarily represent those of their affiliated organizations, or those of the publisher, the editors and the reviewers. Any product that may be evaluated in this article, or claim that may be made by its manufacturer, is not guaranteed or endorsed by the publisher.
