# Supplementary figures and images for: Similarities and Differences in the Acute-Phase Response to SARS-CoV-2 in Rhesus Macaques and African Green Monkeys
Source: Front Immunol. 2021 Oct 6;12:754642. doi: 10.3389/fimmu.2021.754642 (PMC8527883; doi:10.3389/fimmu.2021.754642)

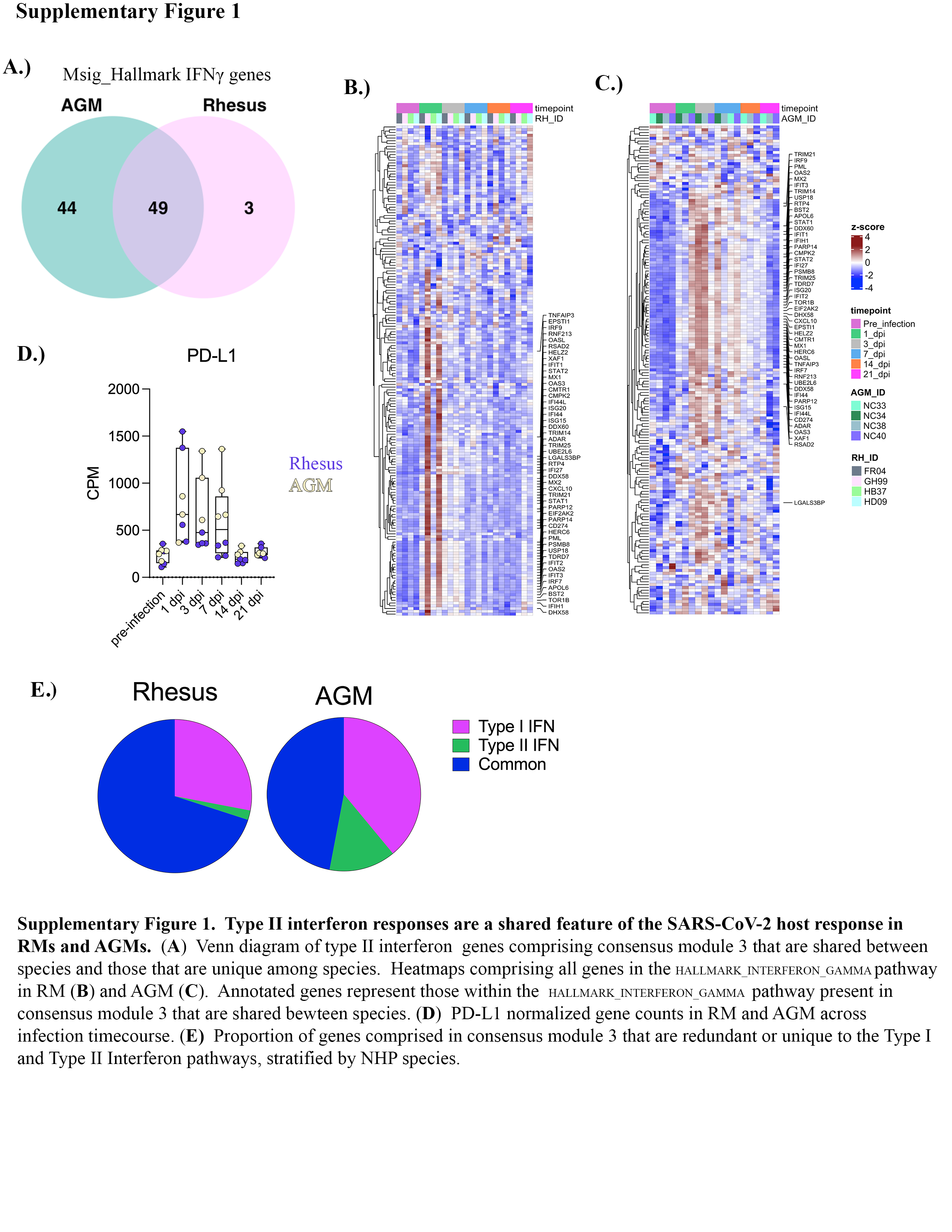

Supplement: Supplementary file 1 [file Image_1.tif]
